# Timing of maternal mortality and severe morbidity during the postpartum period: a systematic review

**DOI:** 10.11124/JBIES-20-00578

**Published:** 2022-08-01

**Authors:** Justine Dol, Brianna Hughes, Mercedes Bonet, Rachel Dorey, Jon Dorling, Amy Grant, Etienne V. Langlois, Joelle Monaghan, Rachel Ollivier, Robin Parker, Nathalie Roos, Heather Scott, Hwayeon Danielle Shin, Janet Curran

**Affiliations:** 1 Faculty of Health, Dalhousie University, Halifax, NS, Canada; 2 Aligning Health Needs and Evidence for Transformative Change (AH_NET-C): A JBI Centre of Excellence, Dalhousie University, Halifax, NS, Canada; 3 School of Nursing, Dalhousie University, Halifax, NS, Canada; 4 UNDP/UNFPA/ UNICEF/WHO/World Bank Special Programme of Research, Development and Research Training in Human Reproduction (HRP), Department of Sexual and Reproductive Health and Research, World Health Organization, Geneva, Switzerland; 5 Division of Neonatal Perinatal Medicine, Department of Pediatrics, Faculty of Medicine, Dalhousie University and IWK Health Centre, Halifax, NS, Canada; 6 Maritime SPOR Support Unit, Halifax, NS, Canada; 7 Partnership for Maternal, Newborn and Child Health, World Health Organization, Geneva, Switzerland; 8 Centre for Research in Family Health, IWK Health Centre, Halifax, NS, Canada; 9 W.K. Kellogg Health Sciences Library, Dalhousie Libraries, Dalhousie University, Halifax, NS, Canada; 10 Clinical Epidemiology Division, Department of Medicine, Karolinska Institutet, Stockholm, Sweden; 11 Department of Obstetrics and Gynecology, Faculty of Medicine, Dalhousie University, Halifax, NS, Canada

**Keywords:** maternal morbidity, maternal mortality, postnatal care, postpartum complications, timing

## Abstract

**Objective:**

The objective of this review was to determine the timing of overall and cause-specific maternal mortality and severe morbidity during the postpartum period.

**Introduction:**

Many women continue to die or experience adverse health outcomes in the postpartum period; however, limited work has explored the timing of when women die or present complications during this period globally.

**Inclusion criteria:**

This review considered studies that reported on women after birth up to 6 weeks postpartum and included data on mortality and/or morbidity on the first day, days 2–7, and days 8–42. Studies that reported solely on high-risk women (eg, those with antenatal or intrapartum complications) were excluded, but mixed population samples were included (eg, low-risk and high-risk women).

**Methods:**

MEDLINE, Embase, Web of Science, and CINAHL were searched for published studies on December 20, 2019, and searches were updated on May 11, 2021. Critical appraisal was undertaken by 2 independent reviewers using standardized critical appraisal instruments from JBI. Quantitative data were extracted from included studies independently by at least 2 reviewers using a study-specific data extraction form. Quantitative data were pooled, where possible. Identified studies were used to obtain the summary estimate (proportion) for each time point. Maternal mortality was calculated as the maternal deaths during a given period over the total number of maternal deaths known during the postpartum period. For cause-specific analysis, number of deaths due to a specific cause was the numerator, while the total number of women who died due to the same cause in that period was the denominator. Random effects models were run to pool incidence proportion for relative risk of overall maternal deaths. Subgroup analysis was conducted according to country income classification and by date (ie, data collection before or after 2010). Where statistical pooling was not possible, the findings were reported narratively.

**Results:**

A total of 32 studies reported on maternal outcomes from 17 reports, all reporting on mixed populations. Most maternal deaths occurred on the first day (48.9%), with 24.5% of deaths occurring between days 2 and 7, and 24.9% occurring between days 8 and 42. Maternal mortality due to postpartum hemorrhage and embolism occurred predominantly on the first day (79.1% and 58.2%, respectively). Most deaths due to postpartum eclampsia and hypertensive disorders occurred within the first week (44.3% on day 1 and 37.1% on days 2–7). Most deaths due to infection occurred between days 8 and 42 (61.3%). Due to heterogeneity, maternal morbidity data are described narratively, with morbidity predominantly occurring within the first 2 weeks. The mean critical appraisal score across all included studies was 85.9% (standard deviation = 13.6%).

**Conclusion:**

Women experience mortality throughout the entire postpartum period, with the highest mortality rate on the first day. Access to high-quality care during the postpartum period, including enhanced frequency and quality of postpartum assessments during the first 42 days after birth, is essential to improving maternal outcomes and to continue reducing maternal mortality and morbidity worldwide.

**Systematic review registration number:**

PROSPERO CRD42020187341

## Introduction

In 2017 alone, approximately 295,000 maternal deaths occurred globally, reflecting a maternal mortality ratio (MMR) of 211 per 100,000 live births across 185 countries.^1^ The global MMR between 2000 and 2017 declined 2.9% per year, on average, with the number of maternal deaths globally in 2017 estimated to be 35% lower than in 2000.^1^ MMRs are significantly higher in low- and middle-income countries (LMICs), with sub-Saharan Africa and southern Asia accounting for 86% of all maternal deaths.^1,2^ Evidence shows that some high-income countries (HICs) are also experiencing an increasing MMR, mostly among vulnerable populations.^3^

However, less is known about timing trends within the immediate 42-day postpartum period to identify when greater follow-up is needed to further reduce mortality. A systematic review of causes of maternal mortality outcomes up until 2012 identified that 73% of maternal deaths were due to direct obstetric causes, such as hemorrhage, hypertensive disorders, and sepsis.^4^ Broadly, 40% to 45% of maternal deaths occur between the start of labor and the 24-hour period immediately after birth.^5^ Much of this evidence focuses on LMICs where the risk of women dying during the postpartum period is significantly higher.^1,2^

Not only is maternal mortality an ongoing concern, but severe maternal morbidity also warrants attention.^6,7^ Various conditions, including, but not limited to, severe postpartum hemorrhage and sepsis are common in the postpartum period.^8^ Severe maternal morbidities are associated with numerous negative short-term and long-term consequences for maternal health and may result in death.^6,7,9^ Severe maternal morbidities highlight the serious complications that can occur during the postpartum period, supporting the need for further investigation into appropriate, timely, and high-quality postpartum care.

Given the growing evidence of severe maternal morbidity and mortality, and the critical role that access to quality care in a timely manner can play in improving outcomes,^10^ it is essential to have access to synthesized information on when deaths and severe morbidities occur during the postpartum period and the range of causes among healthy, low-risk women. After childbirth, a shift occurs from intense monitoring near the end of pregnancy (when women are meeting with health care providers frequently) to significantly reduced care access and utilization.^11^ Existing guidelines in HICs on the timing and frequency of postnatal follow-up care for healthy, low-risk women typically recommend only one visit within a range of 3–8 weeks.^12,13^ Only 2 existing guidelines recommended a visit within the first week (World Health Organization [WHO]^14^ and National Institute for Health and Care Excellence),^15^ with a few guidelines also recommending individualized postnatal follow-up appointments as needed by the woman.^12^

In LMICs, coverage for some essential health care interventions for women (eg, skilled health care providers at birth) has improved, with the global emphasis on the Sustainable Development Goals and support of the Every Woman Every Child initiative.^16^ Still, global estimates suggest that coverage is lower for interventions targeting the postpartum period, with further reductions in coverage for postpartum visits for women compared to newborns.^16^ Understanding when and why mortality and severe morbidity occur in the postpartum period may influence policy and recommendations to enhance coverage of high-quality postnatal care. The current WHO recommendations are for postpartum care to be provided in the first 24 hours after birth at a health facility or within 24 hours if birth took place at home, followed by a minimum of three postpartum contacts occurring within 48 to 72 hours, between days 7 and 14, and 6 weeks after birth.^14^

It is important to ensure the timings for postpartum contact are aligned with when healthy, low-risk women and newborns are experiencing the greatest health challenges in the postpartum period. In light of this, the WHO is currently in the process of updating the Recommendations on Postnatal Care of the Mother and Newborn, as existing guidelines were published in 2013.^14^ Therefore, there is a need to review the literature to identify the timing and causes of maternal and neonatal mortalities and severe morbidities to inform global recommendations. The focus of this review is on maternal mortality and severe morbidities; a second, separate review will focus on neonatal mortality and severe morbidities.

A preliminary search of PROSPERO, MEDLINE, the Cochrane Database of Systematic Reviews, and the *JBI Database of Systematic Reviews and Implementation Reports* was conducted and no current or in-progress systematic reviews on the overall or causespecific timing of maternal mortality and severe morbidity in the postpartum period were identified. Previous reviews that were identified focused on specific aspects, such as frequency of maternal morbidity,^17^ maternal cause-specific analysis between pregnancy and postpartum,^4^ and maternal and perinatal mortality using institutional data in LMICs.^18^ Given the growing number of reports on this topic and the upcoming update of the WHO postnatal care guidelines, it is important to consolidate existing evidence on maternal mortality and severe morbidity outcomes in the healthy, low-risk maternal population during the postpartum period.^19^ Furthermore, the abovementioned review on maternal mortality by Say and colleagues included a search up to 2012^4^; our review adds insight into maternal mortality within the postpartum period by examining deaths on first day (day 1), days 2–7, and days 8–42, with a search up until 2021. Although there are significant contextual differences across HICs and LMICs and diverse health systems, the high mortality ratio and growing morbidity rate for women remain a global issue, thus warranting a review of this magnitude.

The objective of this review is to determine the timing of overall and cause-specific maternal mortality and severe morbidity in the postpartum period.

## Review questions

What is the timing of overall and cause-specific maternal mortality and severe morbidity in healthy, low-risk women in the postpartum period?

In particular:

i) When do women die within the first 42 days after giving birth?
ii) What are the causes of death in women within the first 42 days after giving birth?
iii) When do women experience severe morbidity within the first 42 days after giving birth (overall and cause-specific)?

## Inclusion criteria

### Participants

The review considered reports that included healthy, low-risk women after vaginal or cesarean birth to 6 weeks (42 days) postpartum, consistent with current WHO definitions.^20^ Studies that reported solely on women who were considered high risk (ie, women who need referral for additional management or specialist care; women with intrapartum complications; or women considered high risk as defined by study authors, such as obesity, or preeclampsia prior to delivery) during the perinatal period, or solely on women who delivered before 37 weeks’ gestation or after 42 weeks’ gestation were excluded. Studies that included low-risk and highrisk women (mixed samples) were included.

### Condition

This review sought to locate existing evidence on the overall and cause-specific timing of maternal mortality and severe morbidity during the postpartum period for low-risk women. Maternal death for this study used the following WHO definition, removing the pregnancy portion: “the death of a woman … within 42 days of termination of pregnancy, irrespective of the duration and the site of the pregnancy, from any cause related to or aggravated by the pregnancy or its management, but not from accidental or incidental causes.”^20^^(p^^156)^ Severe maternal morbidity only included severe, direct morbidities that were reported to have occurred after birth and before the end of the postpartum period (42 days). We excluded morbidities and deaths identified during the antenatal period (eg, gestational diabetes, eclampsia) or intrapartum period (eg, intrapartum hemorrhage). Causes were identified using the International Statistical Classification of Diseases, 10^th^ Revision (ICD-10)^8,21^ or as reported by study authors.

### Context

This review considered studies that identified women who gave birth in a health facility or at home. Included studies must have stated that they followed women up to a minimum of 42 days postpartum and must have reported data on first day (day 1), days 2–7, and days 8–42. First-day mortality was defined as death that occurred on the first day or within 24 hours after childbirth, depending on study definition. Although the significant burden of maternal and newborn mortality occurs in LMICs,^22^ given that the Sustainable Development Goals focus on development for all countries,^23^ no limits were placed on country.

### Outcomes

The primary outcomes for this review were as follows:

- timing of maternal mortality: overall
- timing of maternal mortality: cause-specific
- timing and type of severe maternal morbidity.

Due to lack of reporting, the originally defined secondary outcomes in the review protocol^24^ (timing of rehospitalization/readmission by cause and unscheduled use of health services) are not included in this review.

### Types of studies

This review considered studies that provided prevalence or incidence data for maternal mortality and severe morbidity outcomes. This included, but was not limited to, population studies, facility-based studies, and empirical studies (non-experimental). Civil registration vital statistics and populationbased records as available through accessing ministry of health websites of the 194 WHO Member States^25^ and WHO Mortality Database^26^ were also reviewed. Only quantitative studies reporting on prevalence or incidence data were included; qualitative studies and modeling or estimate data (eg, Bayesian modeling, country-level estimates of mortality or morbidity) were excluded. Relevant systematic reviews were used to identify original studies not captured in the search.^17,18^

## Methods

The systematic review was conducted in accordance with JBI methodology for systematic reviews of prevalence and incidence.^27^ An advisory panel with clinical expertise in the areas of neonatology and obstetrics was established to provide consultation and guidance to the review team throughout all stages of the review. This review was conducted in accordance with an *a priori* protocol.^24^ Although the protocol includes both maternal and neonatal outcomes, the neonatal outcomes are reported separately.

### Search strategy

The search strategy, including all identified keywords and index terms, was adapted for each database and developed by a health librarian, as well as peer-reviewed by a second information specialist (Appendix I). The original search was conducted on December 20, 2019, and was updated on May 11, 2021. A Google Scholar search was carried out between July 2–6, 2020, and was updated June 9–12, 2021, using each of the WHO Member States^28^ and (maternal OR neonatal) AND (mortality OR morbidity) to further identify potential sources. The reference lists of all studies selected for critical appraisal were screened for additional studies.

No language limitations were applied to the searches. Studies published in English, French, and Spanish were eligible for inclusion. All reports published since 2000 on data after 2000 were considered for this review. This cut-off was selected to provide the most up-to-date evidence to be used for the update of the 2013 WHO Recommendations on Postnatal Care of the Mother and Newborn.^14^ Additionally, after the introduction of the Millennium Development Goals in 2000, there was a worldwide shift in measurement of mortality and morbidity, resulting in improved quality of data after this period.^29^ Studies that reported on data both before and after 2000 were included and this is noted in the characteristics of the studies. If data were reported separately by year, data older than 2000 were not included.

The databases searched included MEDLINE ALL (Ovid), CINAHL with Full Text (EBSCO), Web of Science Core Collection, and Embase. Search results were limited to publications since January 1, 2000. Sources of unpublished studies and gray literature included ministry of health websites and the Google Scholar search described previously. Prior work in this area was reviewed for additional studies.^4,30,31^ Due to lack of access to a librarian familiar with the database, we were unable to complete the search in LILACS (BIREME – PAHO/WHO website) as stated in the protocol.

### Study selection

All identified citations were uploaded into Covidence (Veritas Health Innovation, Melbourne, Australia) and duplicates were removed through the Covidence automation tool. Titles and abstracts and full texts were then screened by two independent reviewers (JSD, BH, RD, JM, RO, HDS), with disagreements resolved with a third reviewer (JC, MB, JSD, BH) or discussion. Reasons for exclusion of full-text studies that did not meet the inclusion criteria were recorded (Appendix II).

### Assessment of methodological quality

Eligible studies were critically appraised by two independent reviewers (JSD, BR for the English studies and MB, NR for the French and Spanish studies) using standardized critical appraisal instruments from JBI, as appropriate.^32,33^ Any disagreements that arose were resolved through discussion. The results of critical appraisal are reported in narrative form and in tables. All studies, regardless of methodological quality, were included in data extraction and synthesis.

### Data extraction

Data were extracted from papers included in the review by at least two independent reviewers (BH, RD, JM, RO, NR, MB, HDS) using a data extraction tool developed by the reviewers, which was modified and revised through piloting prior to full data extraction (see Appendix III). Any disagreements between reviewers were resolved with a third reviewer (JSD for the English studies) or through discussion for the French/Spanish studies. Authors of two papers were contacted to request missing or additional data for clarification, but they did not respond and were excluded.^34,35^

### Data synthesis

Due to the analysis approach used, there was a deviation from the protocol where the Stata v.14.0 (Stata Corp LLC, Texas, USA) metaprop command was used to conduct analysis of binomial data^36^ instead of the previously planned RevMan v.5.3 (Copenhagen: The Nordic Cochrane Centre, Cochrane). Random effects models were run to pool incidence proportion for overall maternal deaths for most analyses. The metaprop procedures are designed for analysis of binomial data and can manage proportions that are close to 0. Given this, when there were proportions of 0 (ie, studies with 0 deaths at a certain period or for a certain cause at that period), the Freeman-Tukey double arcsine transformation was used to compute the weighted pooled estimate, which stabilizes the variances and ensures they are included in the meta-analysis.

Timing of maternal mortality was calculated as the maternal deaths during a given period over the total number of maternal deaths known during the whole postpartum period (ie, days 1–42). For timing of cause-specific analysis, the number of deaths due to a specific cause on a specific day or period was the numerator while the total number of women who died due to the same cause over the entire postpartum period was the denominator (eg, number of deaths on day 1 related to infection/total number of women who died due to infection in the 42 days following childbirth).

Due to an insufficient number of studies in each category, subgroup analysis on location of birth (facility vs. home) and type of study (population vs. facility-based) were not possible. Subgroup analysis was conducted based on high-, upper-middle-, lower-middle-, and low-income countries according to the 2021 World Bank classification.^37^ While not in the original protocol, the analysis was split by studies that reported on data collected in or before 2010 (2000–2010) and those that collected from 2011 onward (2011–2020) to reflect the changes in maternal mortality that may have occurred over time. Where statistical pooling was not possible for morbidity, the findings are presented in narrative form.

To be included in this review, studies were required to have data on first day mortality (day 1), days 2–7, and days 8–42. Identified studies were used to obtain the summary estimate (proportion) for each time point, and no estimation or extrapolation occurred for missing time points because there were no missing data. One article reported data from multiple countries individually,^5^ and the findings are reported separately at the country level. Another article reported data from multiple countries combined,^38^ and the findings are reported collectively. All other articles reported on a single country. Hereafter, manuscripts with results from multiple countries are referred to as “studies” although multiple “studies” may have data originating from a single published article.

## Results

### Study inclusion

**Figure 1:**
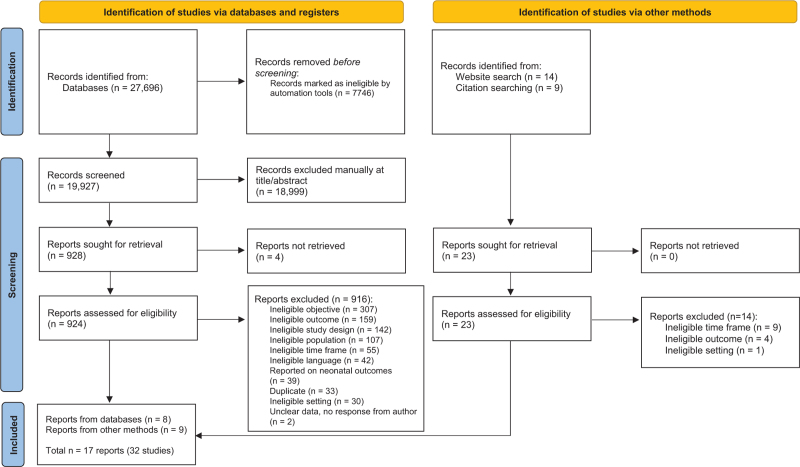
Search results and study selection and inclusion process^39^

Based on the combined search for maternal and neonatal outcomes, 27,673 articles were identified through the original search strategy, and 23 reports were identified through supplementary means (eg, searching reference lists, Google Scholar, ministry of health websites, previously identified systematic reviews). After duplicates were removed through automation tools, 19,927 records were screened using titles and abstracts, after which 18,999 records were excluded. A total of 924 full-text articles were reviewed (4 reports were not able to be retrieved), with 916 excluded for reasons listed in Appendix II. In total, 8 reports were included (see Figure 1^39^). Of the 23 records identified through website and citation searching, 14 were excluded due to irrelevant outcomes, time frames, and settings, and 9 were included. A total of 32 studies from 17 reports were located and included in this review.

The reports were from 23 countries. One article reported on country-level maternal mortality data from seven countries across 10 sites^5^ and another article reported data from seven countries,^40^ with each country considered separately in this analysis (ie, 15 articles reported on data from one country each, and two articles reported on data from a total of 17 country sites = 32 studies).

### Methodological quality

Articles meeting inclusion criteria were critically appraised for methodological quality as appropriate to their study design. Three studies were analytical cross-sectional studies (Table 1),^41-43^ 2 with a score of 100%^42,43^ and one with a score of 63%,^41^ due to unclear reporting on measurement (Q3) and appropriate statistical analysis (Q8), as well as no strategy stated for dealing with confounding factors (Q6). One study was a case-control study with a score of 100% (Table 2).^44^ The prominent type of study design was the cohort study, with 10 studies^5,45-53^ having critical scores ranging from 70% to 100% (Table 3). For this study type, the greatest concerns were whether confounding factors were identified (Q4) and dealt with appropriately (Q5), and whether appropriate statistical analysis was used (Q11). The remaining three articles were prevalence studies,^40,54,55^ with critical appraisal scores ranging from 75% to 88% (Table 4). For prevalence studies, areas where studies scored low included whether the sample was appropriate for the population (Q1), whether the sample size was adequate (Q3), whether the setting/sample were described in detail (Q4), and whether appropriate statistical analysis was used (Q8). Overall, the studies were of sufficient quality, with no studies receiving a score of 60% or below. The mean critical appraisal score was 86% (standard deviation [SD] = 13.6%). Given that the overall critical appraisal scores were high in most of the included studies, this lends credibility to the strength of the findings.

**Table 1:** Critical appraisal of included analytical cross-sectional studies

Citation	Q1	Q2	Q3	Q4	Q5	Q6	Q7	Q8	%
Ferdousy *et al.*^41^ 2018	Y	Y	U	Y	Y	N	Y	U	63
Kingdom of Morocco^42^ 2013	Y	Y	Y	Y	N/A	N/A	Y	Y	100
Kingdom of Morocco^43^ 2010	Y	Y	Y	Y	N/A	N/A	Y	Y	100
%	100	100	67	100	100	0	100	67	

Y, yes; No, no; U, unclear; N/A, not applicable

JBI critical appraisal checklist for analytical cross-sectional studies

Q1. Were the criteria for inclusion in the sample clearly defined?

Q2. Were the study subjects and the setting described in detail?

Q3. Was the exposure measured in a valid and reliable way?

Q4. Were objective, standard criteria used for measurement of the condition?

Q5. Were confounding factors identified?

Q6. Were strategies to deal with confounding factors stated?

Q7. Were the outcomes measured in a valid and reliable way?

Q8. Was appropriate statistical analysis used?

**Table 2:** Critical appraisal checklist for included case-control study

Citation	Q1	Q2	Q3	Q4	Q5	Q6	Q7	Q8	Q9	Q10	%
Acosta *et a!.*^44^ 2014	Y	Y	Y	Y	Y	Y	Y	Y	Y	Y	100

Y, yes

JBI critical appraisal checklist for case-control studies

Q1. Were the groups comparable other than the presence of disease in cases or the absence of disease in controls?

Q2. Were cases and controls matched appropriately?

Q3. Were the same criteria used for identification of cases and controls?

Q4. Was exposure measured in a standard, valid, and reliable way?

Q5. Was exposure measured in the same way for cases and controls?

Q6. Were confounding factors identified?

Q7. Were strategies to deal with confounding factors stated?

Q8. Were outcomes assessed in a standard, valid, and reliable way for cases and controls?

Q9. Was the exposure period of interest long enough to be meaningful?

Q10. Was appropriate statistical analysis used?

**Table 3:** Critical appraisal of included cohort studies

Citation	Q1	Q2	Q3	Q4	Q5	Q6	Q7	Q8	Q9	Q10	Q11	%
AIHW^45^ 2020	Y	Y	Y	U	N	Y	Y	Y	Y	N/A	U	70
AMANHI^5^ 2018	Y	Y	Y	U	Y	Y	Y	Y	U	U	Y	73
Desai *et al.*^46^ 2013	Y	Y	Y	Y	Y	Y	Y	Y	Y	N/A	Y	100
Dossou *et al.*^47^ 2015	Y	Y	Y	U	U	Y	U	Y	Y	N/A	Y	70
Feng *et al.*^48^ 2010	Y	Y	Y	Y	Y	Y	Y	Y	Y	N/A	Y	100
Galambosi *et al.*^49^ 2014	Y	Y	Y	Y	Y	Y	Y	Y	Y	N/A	Y	100
Hacettepe University^50^ 2 0 06	U	N	Y	Y	Y	Y	Y	Y	Y	N/A	Y	80
Iyengar *et al.*^51^ 2009	Y	Y	Y	U	Y	Y	Y	Y	Y	N/A	U	80
Petersen *et al.*^52^ 2019	Y	Y	Y	Y	Y	Y	Y	Y	Y	N/A	U	90
Tang *et al.*^53^ 2 0 09	Y	Y	Y	Y	Y	Y	Y	Y	Y	Y	Y	100
%	90	90	100	70	80	100	90	100	90	50	70	

Y, yes; No, no; U, unclear; N/A, not applicable. AIHW, Australian Institute of Health and Welfare; AMANHI, Alliance for Maternal and Newborn Health Improvement

JBI critical appraisal checklist for cohort studies

Q1. Were the two groups similar and recruited from the same population?

Q2. Were the exposures measured similarly to assign people to both exposed and unexposed groups?

Q3. Was the exposure measured in a valid and reliable way?

Q4. Were confounding factors identified?

Q5. Were strategies to deal with confounding factors stated?

Q6. Were the groups/participants free of the outcome at the start of the study (or at the moment of exposure)?

Q7. Were the outcomes measured in a valid and reliable way?

Q8. Was the follow-up time reported and sufficient to be long enough for outcomes to occur?

Q9. Was follow-up complete, and if not, were the reasons to loss to follow-up described and explored?

Q10. Were strategies to address incomplete follow-up utilized?

Q11. Was appropriate statistical analysis used?

**Table 4:** Critical appraisal of included studies reporting prevalence data

Citation	Q1	Q2	Q3	Q4	Q5	Q6	Q7	Q8	Q9	%
Leonard *et al.*^54^ 2019	Y	Y	Y	N	Y	Y	Y	U	N/A	75
Tepper *et al.*^55^ 2014	U	Y	U	Y	Y	Y	Y	Y	N/A	75
Vousden *et al.*^40^ 2020	U	Y	Y	Y	Y	Y	Y	Y	N/A	88
%	33	100	67	67	100	100	100	67	N/A	

Y, yes; No, no; U, unclear; N/A, not applicable

JBI critical appraisal checklist for studies reporting prevalence data.

Q1. Was the sample frame appropriate to address the target population?

Q2. Were study participants sampled in an appropriate way?

Q3. Was the sample size adequate?

Q4. Were the study subjects and the setting described in detail?

Q5. Was the data analysis conducted with sufficient coverage of the identified sample?

Q6. Were valid methods used for the identification of the condition?

Q7. Was the condition measured in a standard, reliable way for all participants?

Q8. Was there appropriate statistical analysis?

Q9. Was the response rate adequate, and if not, was the low response rate managed appropriately?

### Characteristics of included studies

Twenty-six studies reported data on overall maternal mortality timing, six reported on cause-specific timing outcomes, and seven reported on severe maternal morbidity outcomes. The number of live births or deliveries reported across all mortality studies was 7,704,230 with 6142 maternal deaths. Studies were published between 2006 and 2020 with the time period of data collection between 1996 and 2017. Five studies included data that were collected solely or predominantly in or before 2010, and 21 studies included data collected solely or predominantly from 2011 onward. Fifteen reports were published in English and two were published in French.^42,43^ Most studies reported on a population sample with four studies based on health facilities. The following countries had data from two studies: Bangladesh,^5,41^ Kenya,^5,46^ Morocco,^42,43^ Pakistan,^5^ Tanzania,^5^ United Kingdom,^44,54^ and the United States.^52,55^ with India having data from three studies.^5,51^ Additionally, there were data for each of the following countries: Australia,^45^ China,^48^ Democratic Republic of the Congo,^5^ Ethiopia,^40^ Finland,^49^ France,^47^ Ghana,^5^ Haiti,^40^ Malawi,^40^ Sierra Leone,^40^ Taiwan,^53^ Turkey,^50^ Uganda,^40^ Zambia,^40^ and Zimbabwe.^40^Tables 5 and 6 outline the study characteristics.

**Table 5:** Characteristics of maternal mortality studies

Study/country	Methods	Study population	Live births/ deliveries	Postpartum deaths	Summary of data collection	Results	Maternal inclusion criteria	Limitations/ comments
AMANHI^5^ multi-site	Prospective July 2012–February 2016	Population based Bangladesh, DRC, India, Pakistan, Ghana, Kenya, Tanzania	Bangladesh 26,295 India (H) 35,000 India (U) 37,813 Pakistan (M) 27,062 Pakistan (K) 17,189 DRC 6145 Ghana 23,640 Kenya 30,992 Tanzania (I) 8128 Tanzania (P) 18,882	Bangladesh 103 India (H) 41 India (U) 113 Pakistan (M) 51 Pakistan (K) 62 DRC 24 Ghana 42 Kenya 17 Tanzania (I) 24 Tanzania (P) 53	Verbal autopsy	Day 1 Bangladesh: 48 (46.6%) India (H): 17 (41.5%) India (U): 75 (66.4%) Pakistan (M): 33 (64.7%) Pakistan (K): 41 (66.1%) DRC: 19 (79.2%) Ghana: 20 (47.6%) Kenya: 8 (47.1%) Tanzania (I): 11 (45.8%) Tanzania (P): 32 (60.4%) Days 2-7 Bangladesh: 17 (16.5%) India (H): 10 (24.4%) India (U): 17 (15.0%) Pakistan (M): 9 (17.6%) Pakistan (K): 7 (11.3%) DRC: 2 (87.5%) Ghana: 6 (14.3%) Kenya: 0 (0%) Tanzania (I): 7 (29.2%) Tanzania (P): 7 (13.2%) Days 8-42 Bangladesh: 38 (36.9%) India (H): 1434.1%) India (U): 21 (18.6%) Pakistan (M): 9 (17.6%) Pakistan (K): 15 (24.2%) DRC: 3 (12.5%) Ghana: 16 (38.1%) Kenya: 9 (52.9%) Tanzania (I): 6 (25%) Tanzania (P): 14 (26.4%)	Pregnant women of reproductive age (15–49 years), followed from birth to 42 days postpartum	–
Australian Institute of Health and Welfare^45^ Australia	Cohort study 2015–2017	Population based	915,610	128	National data	Day 1: 40 (31.2%) Days 2-7: 29 (22.7%) Days 8-42: 59 (46.1%) Weekly	Death of a woman while pregnant or within 42 days of the end of pregnancy, irrespective of the duration and outcome of the pregnancy, from any cause related to or aggravated by the pregnancy	—
Desai *et al.*^46^ Kenya	Cohort study, case control January 2003–December 2008	Population based Nyanza Province	NR	103	Verbal autopsy; Health and Demographic Surveillance System	Day 1: 37 (36.0%) Days 2-7: 33 (32.0%) Days 8-42: 33 (32.0%)	All female residents aged 15–49 years at the time of death	Overall mortality
Feng *et al.*^48^ China	Cohort study 1996-2006	Population based Beijing, Shanghai, Tianjin regions	6,253,008	2347	National data	Day 1: 1592 (67.8%) Days 2-7: 409 (17.4%) Days 8-42: 346 (14.7%) Weekly	No limitations	Includes data collected prior to 2000 Overall and cause-specific mortality
Hacettepe University Institute of Population Studies^50^ Turkey	Cohort study October 2004–December 2006	Population based Various regions in Turkey	763,585	158	Health center data; verbal autopsy; national data; cemetery burial list	Day 1: 61 (38.6%) Days 2-7: 43 (27.2%) Days 8-42: 54 (34.2%)	All women of reproductive age 15–49 years	Multiple gestations included in sample
Iyengar et ol.^51^ India	Cohort study June 2002–May 2003	Population based Southern Rajasthan region	4648	24	Verbal autopsy	Day 1: 7 (29.2%) Days 2-7: 8 (33.3%) Days 8-42: 9 (37.5%)	Pregnancy-related deaths of women aged 15–49 years	Overall and cause-specific mortality
Kingdom of Morocco 2010^43^ Morocco	Cross-sectional January–December 2009	Population based	NR	225	Health center data; verbal autopsy; national data	Day 1: 142 (63.1%) Days 2-7: 55 (24.4%) Days 8-42: 28 (12.4%)	Maternal death cases with completed reports, occurring during pregnancy and 42 days after birth	Overall and cause-specific mortality
Kingdom of Morocco 2013^42^ Morocco	Cross-sectional January–December 2010	Population based	NR	210	Health center data; verbal autopsy; national data	Day 1: 133 (63.3%) Days 2-7: 55 (26.2%) Days 8-42: 22 (10.5%)	Maternal death cases with completed reports, occurring during pregnancy and 42 days after birth	Overall and cause-specific mortality
Petersen *et al.*^52^ United States	Retrospective 2011–2015 and 2013–2017	Population based	NR	1702	National data; death certificates	Day 1: 506 (29.7%) Days 2-7: 556 (32.7%) Days 8-42: 640 (37.6%)	All women who died during pregnancy or within 1 year after delivery	Overall and cause-specific mortality
Vousden *et al.*^40^ Multi-site	Secondary analysis of randomized controlled trial April 2016–November 2017	Population based Zimbabwe, Zambia, Sierra Leone, Malawi, Ethiopia, Uganda, Haiti, and India	Ethiopia 35,429 Haiti 14,910 India 22,876 Sierra Leone 23,806 Malawi 62,165 Uganda 188,319 Zambia 150,345 Zimbabwe 38,383	Ethiopia 29 Haiti 23 India 25 Sierra Leone 99 Malawi 118 Uganda 289 Zambia 80 Zimbabwe 52	Health center data; national data	Day 0 Ethiopia: 13 (44.8%) Haiti: 13 (56.5%) India: 5 (20%) Sierra Leone: 61 (61.6%) Malawi: 57 (48.3%) Uganda: 168 (58.1%) Zambia: 31 (38.8%) Zimbabwe: 8 (15.4%) Days 1-6 Ethiopia: 10 (34.5%) Haiti: 9 (39.1%) India: 7 (28%) Sierra Leone: 28 (28.3%) Malawi: 34 (28.8%) Uganda: 100 (34.6%) Zambia: 31 (38.8%) Zimbabwe: 29 (55.8%) Days 7-42 Ethiopia: 6 (20.7%) Haiti: 1 (4.3%) India: 13 (52%) Sierra Leone: 10 (10.1%) Malawi: 27 (22.9%) Uganda: 21 (7.3%) Zambia: 18 (22.5%) Zimbabwe: 15 (28.8%)	All women who were recorded as having died at any gestation or up to 42 days after delivery, from any cause	—

DRC, Democratic Republic of Congo; H, Haryana; I, Ifakara; K, Karachi; M, Matiari; NR, not reported; P, Pemba; U, Uttar Pradesh

**Table 6:** Characteristics of maternal morbidity studies

Study/country	Methods	Study population	Live births/ deliveries	Postpartum deaths	Summary of data collection	Morbidity focus	Maternal inclusion criteria	Limitations/ comments
Acosta *et al.*^44^ United Kingdom	Prospective case-control June 2011–May 2012	Population based England, Northern Ireland, Scotland, Wales and Crown Dependencies	780,537	NR	National data, United Kingdom Obstetric Surveillance System	Severe sepsis	Women who gave birth to a live or stillborn infant of greater than 24 completed weeks of gestation	—
Dossou *et al.*^47^ France	Retrospective January 2004–February 2013	Facility-based	26,023	NR	Health center data	Late postpartum hemorrhage	All women who gave birth at the Clermont-Ferrand University Hospital Center (level III) and who had severe secondary postpartum hemorrhage	—
Ferdousy *et al.*^41^ Bangladesh	Cross-sectional January 2016–December 2016	Facility-based	NR	NR	Health center data	Late postpartum hemorrhage	All patients admitted in Rangpur Medical College Hospital with a diagnosis of secondary postpartum hemorrhage over a period of 1 year	—
Galambosi *et al.*^49^ Finland	Cohort study 2001–2011	Population based	634,292	NR	National data	Venous thromboembolism	Women with an inpatient or outpatient admission after date of delivery with a diagnosis of venous thromboembolism	—
Leonard *et al.*^54^ United Kingdom	Retrospective, cross-sectional January 2010–December 2016	Population based London and Southeast regions	1,598,069	NR	Health center data	Maternal Group A streptococcal infection	All laboratory- confirmed invasive Group A streptococcus cases in women in London and the Southeast of England with a date of onset within 28 days of birth	—
Tang *et al.*^53^ Taiwan	Cohort study 1999–2003	Facility-based	NR	NR	National data	Stroke	Women where the birth certificate dataset and delivery entries in the NHI hospital discharge data were successfully linked	Includes data prior to 2000
Tepper *et al.*^55^ United States	Retrospective 2005–2011	Facility-based Commercial and Medicaid databases	2,542,562	NR	National data	Venous thromboembolism	Women aged 15–44 years with information on pharmaceutical claims who had a delivery hospitalization	—

NHI, national health insurance; NR, not reported.

### Review findings

#### Timing of overall postpartum maternal mortality

**Figure 2:**
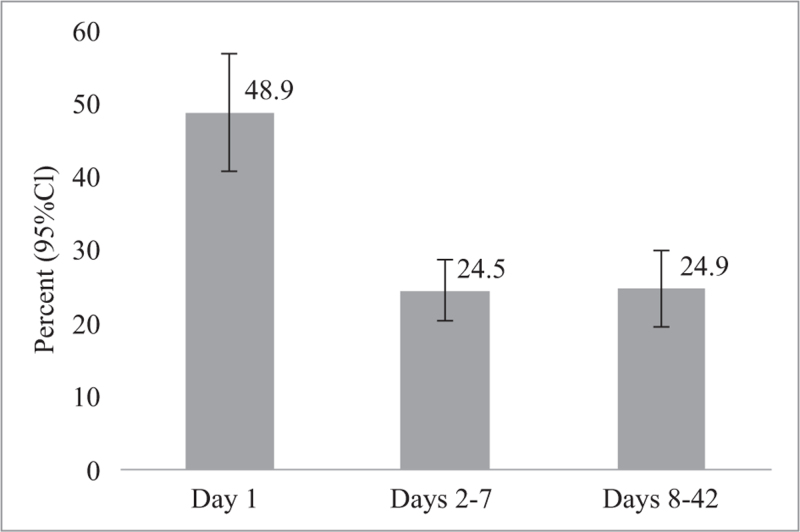
Proportion of postpartum maternal deaths on day 1, days 2–7, and days 8–42 based on data from 26 studies including 8,704,230 women and 6142 maternal deaths; see Appendix IV for confidence intervals

**Figure 3:**
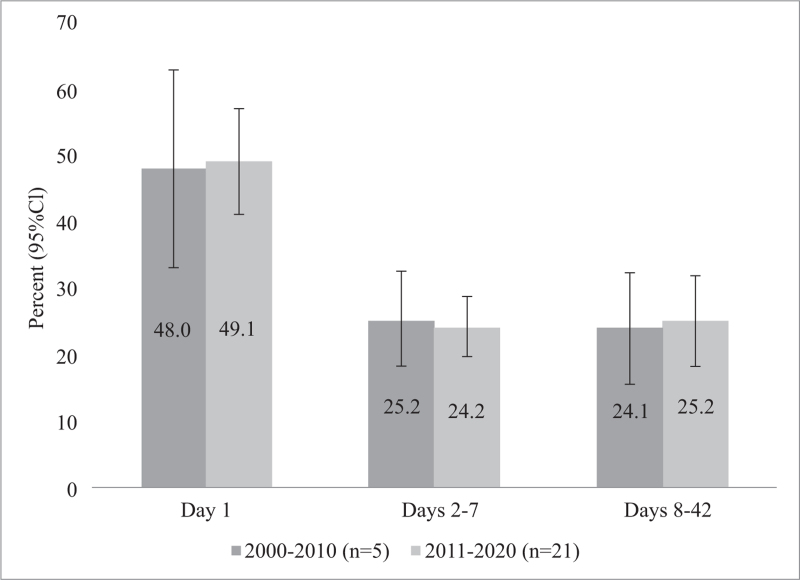
Proportion of postpartum maternal deaths on day 1, days 2–7, and days 8–42 between 2000 and 2010 (n = 5 studies, 7,021,214 women) and between 2011 and 2020 (n = 21 studies, 1,682,989 women)

Based on data from 26 studies including 8,704,230 women, most postpartum maternal deaths occur on day 1 (48.9%), with 24.5% of deaths between days 2 and 7, and 24.9% between days 8 and 42 (see Figure 2). See Appendix IV for confidence intervals. These proportions remain consistent when considering studies that report on data from or before 2010 and 2011 onward (see Figure 3).

**Figure 4:**
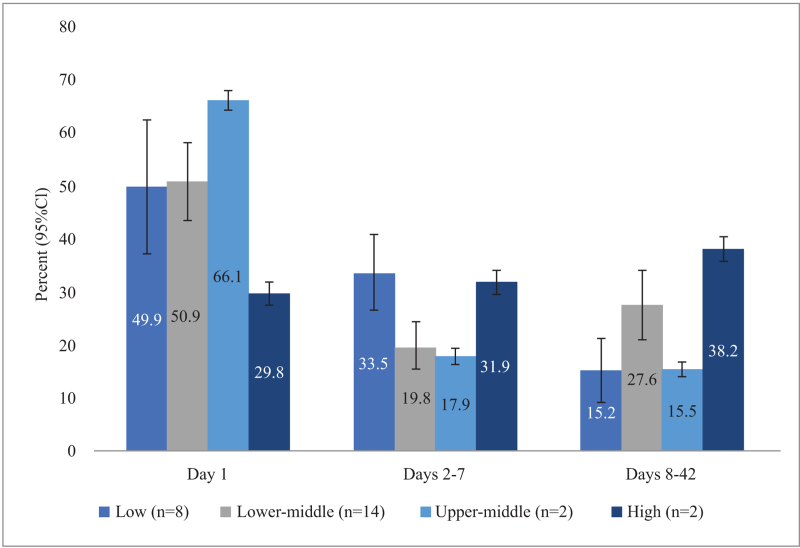
Proportion of postpartum maternal deaths by country income level (number of studies) on day 1, days 2–7, and days 8–42; see Appendix IV for confidence intervals

When considering postpartum maternal mortality by country income classification, timing of maternal deaths varies (Figure 4). For low-income countries (n = 8 studies; 519,502 women) and lower-middle-income countries (n = 14 studies; 252,525 women), the proportion of maternal deaths is similar on day 1 (49.9% and 50.9%, respectively); however, there are differences in the distribution of subsequent deaths. In low-income countries, the proportion of deaths decreased over the postpartum period, with fewer occurring on days 2–7 (33.5%) and days 8-42 (15.2%). In lower-middle-income countries, more deaths occurred in days 8–42 (27.6%) than in days 2–7 (19.8%). For upper-middle-income countries (n = 2 studies, 7,955,078 women), a higher proportion of deaths occur on day 1 (66.1%) compared with other country-level income groups. High-income countries (n = 2 studies, 915,610 women) have the lowest proportion of deaths on day 1 at 29.8% and the highest between days 8–42 at 38.2%. See Appendix IV for confidence intervals.

#### Timing of cause-specific postpartum maternal mortality

**Figure 5:**
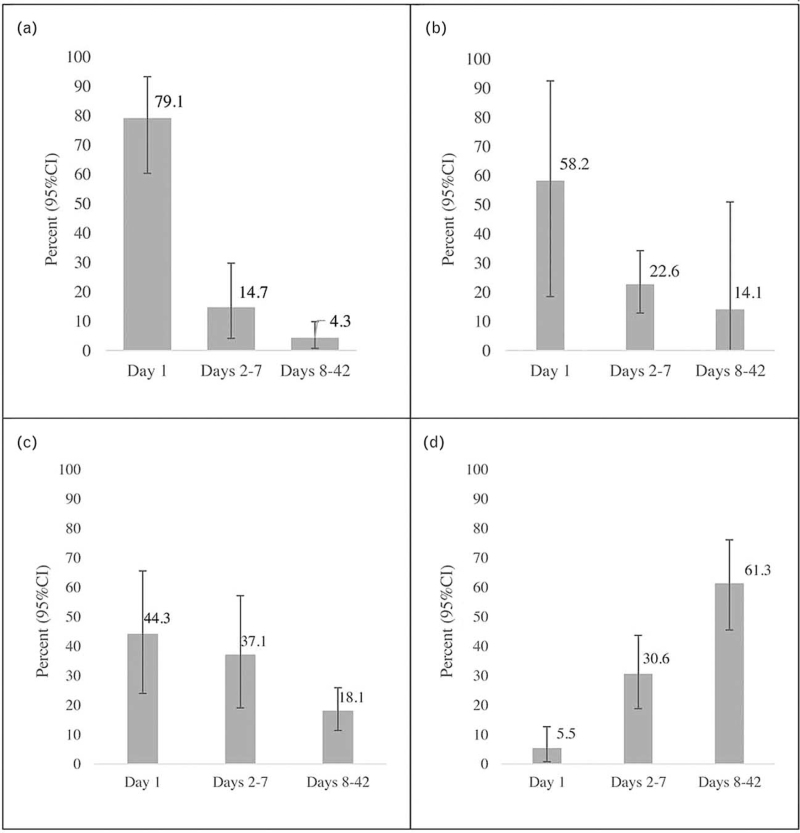
Proportion of postpartum maternal mortality on day 1, days 2–7, and days 8–42 due to (a) postpartum hemorrhage (n = 6 studies, 1561 deaths); (b) embolism (n = 3 studies, 408 deaths); (c) postpartum eclampsia/hypertensive disorders (n = 4 studies, 338 deaths); and (d) infection (n = 6 studies, 840 deaths); see Appendix IV for confidence intervals

Seven studies reported on cause-specific timing of postpartum maternal mortality. Of the total 6142 maternal deaths identified, causes were available for 2727 deaths (44.4%). Based on the causes reported, timing of maternal mortality was grouped by postpartum hemorrhage, embolism (eg, amniotic fluid embolism, thrombotic pulmonary, other embolism), infection (eg, sepsis and not specified), and eclampsia/hypertensive disorders. Of note, these deaths occurred in the postpartum period, but the onset of the cause leading to death may have been in the antenatal/intrapartum period. As shown in Figure 5, with confidence intervals in Appendix IV, maternal mortality due to postpartum hemorrhage and embolism occurred predominantly on day 1 (79.1% and 58.2%, respectively). Postpartum deaths due to eclampsia/hypertensive disorders occurred mainly in the first week, with 44.3% of deaths on day 1 and 37.1% of deaths on days 2–7. Deaths due to infection were more likely to occur between days 8–42 (61.3%) followed by days 2–7 (30.6%). Due to the small number of studies for each cause, no subgroup analysis was possible.

#### Timing and causes of severe postpartum maternal morbidity

Seven studies reported on severe postpartum maternal morbidity outcomes, with these reported narratively due to heterogeneity across outcomes.

Acosta and colleagues^44^ reported on severe sepsis, with no specified source and inclusive of septic shock, in women who had obstetrician-assisted births in health centers across the United Kingdom. Between 2011 and 2012, the authors reported a median time between birth and development of maternal sepsis of 3 days (interquartile ratio [IQR] 1–7 days). Critical care was required for 79.2% of postpartum women and septic shock occurred in 23.8%. Leonard and colleagues^54^ examined the impact of severe maternal invasive group A streptococcal (iGAS) disease in women from England (97.8% postpartum) between 2010 and 2016. A total of 134 maternal iGAS cases were identified, of which 87% occurred in the first week postpartum, 30.0% on the day of birth, 57.0% between days 2 and 7, and 13% between 8 and 28 days postpartum. Median onset time occurred two days after birth with an IQR of 0–5 days.

Galambosi and colleagues^49^ explored the incidence of venous thromboembolism (VTE), including deep vein thrombosis, portal vein thrombosis, and pulmonary embolism, in postpartum women from Finland over a 10-year period (2001–2011). This study included a mixed sample of women, with some identified as high-risk for having pre-existing conditions, or pregnancy or intrapartum complications. The study reported the timing of VTE diagnosis as an aggregate of the entire sample. A total of 1169 VTEs out of 634,292 deliveries were found in postpartum women (age range: 15–49 years), of which 16.4% had a cesarean birth. VTE was reported weekly, with 425 diagnoses noted in week 1 (36.4%), 29 in week 2 (2.5%), 44 in week 3 (3.8%), 37 in week 4 and 5 (3.2%), and 33 in week 6 (2.8%). Tepper and colleagues^55^ examined postpartum VTE incidence from the United States in 2005–2011, comparing women with private insurance (n = 1,540,026) versus Medicaid (n = 1,002,536). Timing data related to VTEs was reported overall, with a total of 4169 diagnoses across both groups (35.4% cesarean birth). There were 2359 diagnoses reported in week 1 (56.6%), 621 in week 2 (14.9%), 382 in week 3 (9.2%), 245 in week 4 (5.9%), 154 in week 5 (4.0%), and 92 in week 6 (2.2%). Tepper and colleagues^55^ noted that increased age was found to be a risk factor only in early diagnoses of VTE (eg, first week).

Dossou and colleagues^47^ reported on postpartum hemorrhage (PPH) over a nine-year period (20042013) in women from a level-III health facility in France, and in particular, secondary PPH, which is defined as severe bleeding after 24 hours of birth and up to 42 days. Women included in the sample were 30.4 years of age on average (SD = 5.7), and modes of birth included 66.7% spontaneous vaginal, 8.3% operative vaginal (eg, forceps), and 25% cesarean birth. The average onset of PPH was 13.4 days postpartum (SD = 10.8), ranging from 1–39 days after birth, with 86.7% of PPH occurring at home and requiring readmission.^47^ Ferdousy and colleagues^41^ also reported on secondary PPH among women in Bangladesh readmitted to the hospital. The mean age of women was 25.2 years (SD = 2.8), and 56% gave birth through a cesarean delivery. The mean time of presentation for secondary PPH was 13.5 days (SD = 2.8), with 47% of cases occurring during the second week and 34% occurring during the third week.^41^

Tang and colleagues^53^ reported on cases of stroke (hemorrhagic and ischemic) in postpartum Taiwanese women between 1999 and 2003. During the study period, 1,136,477 live births were recorded, of which there were 243 stroke cases (15.6% prenatal; one pregnancy-related hypertension). In the first 3 days postpartum, 32 cases of stroke were identified, and another 34 cases were reported between days 4 and 42 postpartum. Among the population who had a stroke, postpartum hemorrhage occurred within 3 days after birth (n = 2) and between 4 days and 6 weeks postpartum (n = 1). Pre-eclampsia diagnoses were also found in women experiencing postpartum stroke (n = 47 within 3 days; n = 3 between days 4 and 42).

## Discussion

The objective of this review was to determine the timing of overall and cause-specific maternal mortality and severe morbidity in women during the postpartum period (days 1 through 42 after childbirth). Overall, we found that day 1 has the largest proportion of postpartum maternal deaths (48.9%), with 24.5% of deaths occurring between days 2 and 7, and 24.9% between days 8 and 42. This remained consistent when exploring data published before 2010 or after. In consideration of the timing of women's death during the postpartum period, the first day is a critical period for women's survival, which may be influenced by causes that originated during the antenatal or intrapartum period. However, one-fourth of women die between days 8 and 42, suggesting continued care throughout the first 42 days after childbirth is important for improving maternal outcomes. In terms of causes, hemorrhage and embolism remain of great concern on the first day, with postpartum eclampsia and hypertension also causing concerns in the first week. Infection was the leading cause of death for days 8–42. Due to heterogeneity, no specific conclusions could be drawn related to timing of severe maternal morbidity.

This is the first review to our knowledge that examined the timing of maternal mortality and severe morbidity in the postpartum period worldwide using both population and facility-based studies. Much of the previous work has focused on projections or mortality within the postpartum period as a whole, without any specific analysis of timing in the postpartum period or focused on timing across the perinatal period broadly (ie, mortality during pregnancy, intrapartum, and postpartum). Our work seeks to fill the gap in mortality and severe morbidity timing during the postpartum period.

### Overall postpartum maternal mortality

The highest mortality rate was on the first day postpartum for women living in low-income, lower-middle-income, and upper-middle-income countries. However, the country income classification analysis needs to be interpreted with caution because of the small number of studies in uppermiddle- and high-income countries, and scarce data on timing of cause-specific mortality, with less than half of deaths having an associated cause. Previous work has shown that the least developed countries have a higher overall maternal absolute mortality ratio than more developed countries.^1^ Additionally, Merdad and Ali^56^ examined maternal mortality during the entire perinatal period in 34 sub-Saharan African countries and found significant variability between countries; however, it has been estimated that sub-Saharan Africa accounts for 66% of maternal mortality.^1^ This is why it is important to consider the country income classification analysis, as an overall proportion may not reflect the variation of timing in different countries, particularly in low- income and lower-middle-income countries, where the predominant burden of maternal mortality exists.^57^

HICs had the lowest proportion of deaths on the first day but had the highest pooled incidence of mortality between days 8 and 42, suggesting that women in HICs are more likely to die later in the postpartum period compared with the first week. This may be related to the likelihood that first-day deaths could be highly correlated with intrapartum causes.^56^ Women in HICs are more likely to have access to high-quality antenatal care and to give birth at a health facility or in the presence of skilled health care providers,^58^ thus they are able to receive the lifesaving care they need during pregnancy and on the first day postpartum. Women in HICs also have access to intensive care units where they are able to get rapid care and may be able to survive longer than in lower-resource settings. However, because data are limited relating to when the condition was diagnosed, when death occurred, and when each women had antenatal and postnatal contacts, it is difficult to delineate the true reasons. Additionally, because the data are from only a few studies, this review was unable to compare cause-specific analysis at the country income level to allow investigation of potential differences in causes of death. Nevertheless, high-quality, continued care throughout the postpartum period is necessary to reduce maternal mortality, regardless of country.

### Cause-specific postpartum maternal mortality

Out of a range of potential causes of maternal mortality in women during the postpartum period, postpartum hemorrhage, embolism, and postpartum eclampsia/hypertensive disorders remain of great concern in the first week, as well as infection between days 8 and 42 postpartum period. In the interpretation of cause-specific maternal mortality, it is important to consider that women may have other morbidities or mortalities that were not reported if no study on timing was identified. Additionally, some of the causes of mortality may be the result of events that occurred during pregnancy or childbirth, but the death occurred during the postpartum period. Thus, it is important to consider the women throughout the perinatal period in order to improve outcomes during the postpartum period.

Consistent with our review, a recent evaluation of cause-specific maternal mortality globally found hemorrhage to be the most common cause of death, of which two-thirds of hemorrhage-related deaths occurred postpartum.^4^ Hemorrhage has steadily been recognized as a leading contributor to maternal mortality^56^ despite the advancement of clinical interventions to manage this complication.^4^ While the number of hemorrhagic deaths was reported to vary globally, with greatest incidence of death noted in northern Africa,^4^ our review was not able to conduct this level of analysis with the available data. However, hemorrhage remains the leading cause of death across all regions,^4^ thus warranting attention worldwide. Greater prevention and management strategies in the postnatal period and improved reporting of implementation strategies to evaluate the effectiveness of interventions are needed.^3^

Additionally, postpartum eclampsia was in the top three causes of mortality across the entire postpartum period. Pre-eclampsia and eclampsia during pregnancy are common yet serious diagnoses, with these also being leading causes of maternal death globally.^4,59,60^ Matthys and colleagues^61^ evaluated all pre-eclampsia and eclampsia diagnoses over a 10year period, finding that 5.7% of diagnoses occurred during the postpartum period. They also noted that women readmitted with postpartum pre-eclampsia and eclampsia presented symptoms that were easily attributed to normal postpartum physical adjustment symptoms, such as headache or abdominal pain, for which they did not seek timely medical care.^61^ Signs and symptoms of postpartum preeclampsia present similarly to pre-eclampsia during pregnancy and require prompt treatment,^61,62^ yet the frequency of monitoring and access to quality care is greatly reduced in the postpartum period.^10^ Thus, improved discharge education for families regarding self-monitoring of symptoms^62^ and enhanced access to quality health care from birth up to 42 weeks postpartum is necessary.

Between days 8 and 42 postpartum, causes of mortality shifted to infection as the main cause. Although timing was not specified in previous findings, sepsis in the postpartum period was documented as a leading cause of death, ranking third globally^4^ and second throughout 34 sub-Saharan African countries.^56^ Say and colleagues^4^ noted that most sepsis- related death occurred in LMICs, but recent evaluations found that postpartum infections were concerning in HICs including the United States^63^ and the United Kingdom.^44^ Contributing factors related to postpartum infection include cesarean or operative vaginal births,^64,65^ limited knowledge of signs of infection,^66^ barriers to timely access to health care providers,^66^ and appropriate use of antibiotics.^67^ Given the worldwide rise in cesarean deliveries,^68^ we can anticipate there may be a corresponding increase in the number of postpartum infections. Although cesarean birth may be a necessary intervention for both the woman and her newborn, the associated risk of infection must be taken into account and anticipated.^69^ Access to quality postpartum services with appropriate treatment specific to the source of infection is essential in reducing postpartum mortality between days 8 and 42.

### Severe postpartum maternal morbidity

In our review, seven studies reported on a range of morbidity outcomes, which limited our ability to synthesize the findings. However, across all morbidity conditions, the predominant onset across all morbidities reported tends to occur in week 1, followed by week 2. For instance, in this review, we found that VTE occurred within the first week between 36.4%^49^ and 56.6%^55^ of the time, and secondary PPH had an average onset of 13 days.^41,47^ Previous work has found that severe maternal morbidity trends are similar to maternal mortality trends, with higher rates in LMICs compared with HICs.^70^ In a recent multi-site cohort study on maternal morbidity of 735,000 women, 32.7% of pregnancies had at least one experience of maternal morbidity, with women in South Asia experiencing higher morbidities (43.9%) than women in subSaharan Africa (23.7%).^71^ In addition, previous work has suggested that 8% of hospital deliveries in LMICs are complicated by a severe morbidity, caused mostly by hemorrhage, hypertension, and sepsis.^17,70-72^ While the first day was also found to be of high incidence for morbidity, concerns continued beyond the postpartum period with infections being identified within two to three days after birth.^44,54^ All of the data points in this review represent only one study in one location, and thus must be interpreted with caution. However, the combined data show that the first two weeks pose a high potential for maternal postpartum morbidity outcomes, with the first week and first day being a particularly vulnerable time, depending on the morbidity identified.

It is important for women to know the danger signs of potential severe morbidity and have access to health services. Additionally, discharge and postpartum education and counseling should focus on potential risks and symptoms so that women can monitor their physical health after birth, understand what is normal or abnormal, and seek help in a timely manner. Previous work has identified barriers to the uptake of postpartum health service; these barriers are linked to low awareness by women and family members regarding signs of postpartum complications as well as a hesitancy to contact health workers due to concerns around trust and poorquality care.^73,74^ Previous work also has identified challenges of accessing quality postnatal care, including health care provider competencies, low quality of care, and inability of the health care system to provide adequate postnatal care.^10^ Thus, providing discharge education targeting women and their families related to the importance of postnatal health care utilization may be an approach to enhance usage, as well as improving the training and support for health care providers to enhance the quality of postnatal care for women and their newborns.

### Limitations

While this review is the first to our knowledge to examine specifically when women die or experience severe morbidity in the postpartum period worldwide, there are several limitations. First, we only used data as provided in identified studies. Although this adds strength in that no estimation or projection is used on the data, there may be over- or underestimation of mortality based on the country and type of study published. There is also high heterogeneity across the studies, which could be attributed to many factors, including study type as well as variation in geographical locations, measurement approaches, or access to health facilities. Additionally, we included both population- and facility-based studies in this review, which may result in bias in terms of incomplete follow-up; however, all included studies must have reported that they followed all participants up to 42 days, likely limiting this potential source of bias in the analysis. These limitations should be taken into consideration when interpreting these results.

A second limitation is the difficulty associated with reporting maternal mortality, both in timing and causes, with many deaths being uncounted and many countries experiencing challenges reporting mortality, both in terms of completeness and misclassification.^1^ When women die at home, which is often the case in the postpartum period, particularly in low-resource settings, it is difficult to capture the exact timing and cause, and the use of verbal autopsy has limitations for data collection.^2^ Given that almost all of the included studies were populationbased, this is reflected in our data. Reporting of timing in relation to cause of death is a limitation found in our synthesis, with only 7 studies providing this data. Studies may have difficulty determining mortality causes or specific timing in low-resource areas where there is limited ability to conduct follow-up evaluation (eg, autopsies)^22^ and inaccurate reporting of causes.^4^

A third limitation of this review is our focus only on physical severe morbidities, which excludes the mental health complications as well as incidental causes, such as intimate partner violence and other factors that have been associated with negative health outcomes for women post-birth.^17,75^ There is emerging evidence of the role that non-communicable diseases and maternal suicide has in maternal mortality and morbidity rates.^76,77^ We also included mortality and morbidity outcomes only within the first 42 days but did not examine late maternal mortality outcomes (43 days to one year postpartum).

A final limitation is that we were not able to explore differences in mortality and morbidity due to differences in type of birth (eg, vaginal vs cesarean delivery) or parity, as this was not reported at this level in the identified studies. However, it is possible that differences exist in mortality and morbidity estimates based on these factors. Future work should consider reporting on timing of maternal mortality and morbidity based on these factors.

## Conclusions

Women are at risk of mortality across the entire postpartum period, with the highest mortality rate on the first day. Enhancing the frequency and quality of postpartum contacts during the first 42 days may improve maternal outcomes while reducing maternal mortality and severity of morbidities during this time period.

### Recommendations for practice

Given the maternal and severe morbidity incidence for women over 42 days postpartum, and in particular, during the first day postpartum, this review supports the need for postpartum contacts across the entire period. Existing guidelines recommend a minimum of 4 postnatal care contacts.^14^ Because of the critical nature of the outcomes and significant number of deaths occurring across the entire postpartum period in both absolute and relative terms, increasing frequency and quality of postpartum visits for healthy women should be considered. Government support and training of health care providers related to postnatal care for women and their newborn is important, including the ongoing assessment of competencies, a healthy work environment, and the resources necessary to provide quality postnatal care.

### Recommendations for research

Further research should focus on collecting timespecific data on maternal mortality and severe morbidity, particularly in low-income countries, reporting both overall and cause-specific daily and weekly deaths. While this is notoriously harder to do as a result of reporting challenges in health facilities and when women die at home, it is important that future research and registration data attempt to capture this information to truly understand when women are dying during the postpartum period. Additionally, it would be important to map the timing of mortality and morbidity against postnatal contact coverage in different countries across the postnatal period. More specific reporting of morbidity onset is recommended to help identify the greatest risk factor in terms of timing for healthy, low-risk women in the postpartum period.

## Acknowledgments

Ann-Beth Moller for providing input into the protocol and commenting on the manuscript.

## Funding

This project was supported with funding in part by the Canadian Institutes of Health Research (CIHR) under the Strategy for Patient Oriented-Research (SPOR) initiative through the SPOR Evidence Alliance and the UNDP/UNFPA/UNICEF/WHO/World Bank Special Programme of Research, Development and Research Training in Human Reproduction (HRP), Department of Reproductive Health and Research, World Health Organization, Geneva, Switzerland. The funders did not have any role in content development.

## Appendix I: Search strategy

### Ovid MEDLINE

Search conducted on December 12, 2019 with 8442 studies identified; updated on May 10, 2021 with 1289 studies identified

### Embase

Search conducted on December 20, 2019 with 3400 studies identified; updated on May 10, 2021 with 842 studies identified.

### Web of Science

Search conducted on December 20, 2019 with 6151 studies identified; updated on May 10, 2021 with 512 studies identified.

### CINAHL (EBSCO)

Search conducted on December 20, 2019 with 4058 studies identified; updated on May 10, 2021 with 217 studies identified.

((((((TI (((After OR following) N2 (birth^∗^ OR deliver^∗^)) N2 (complication^∗^ OR morbidit^∗^ OR mortalit^∗^ OR death^∗^ OR hemorrhag^∗^ OR haemorrhag^∗^ OR bleed^∗^ OR anaemi^∗^ OR anemi^∗^ OR infected OR infection^∗^ OR sepsis OR septic))) OR (AB (((After OR following) N2 (birth^∗^ OR deliver^∗^) N2 (complication^∗^ OR morbidit^∗^ OR mortalit^∗^ OR death^∗^ OR hemorrhag^∗^ OR haemorrhag^∗^ OR bleed^∗^ OR anaemi^∗^ OR anemi^∗^ OR infected OR infection^∗^ OR sepsis OR septic))))) OR ((TI (((Postnatal^∗^ OR post natal^∗^ OR post partum OR postpartum OR puerperal) N2 (complication^∗^ OR morbidit^∗^ OR mortalit^∗^ OR death^∗^ OR hemorrhag^∗^ OR haemorrhag^∗^ OR bleed^∗^ OR anaemi^∗^ OR anemi^∗^ OR infected OR infection^∗^ OR septic OR sepsis)))) OR (AB (((Postnatal^∗^ OR post natal^∗^ OR post partum OR postpartum OR puerperal) N2 (complication^∗^ OR morbidit^∗^ OR mortalit^∗^ OR death^∗^ OR hemorrhag^∗^ OR haemorrhag^∗^ OR bleed^∗^ OR anaemi^∗^ OR anemi^∗^ OR infected OR infection^∗^ OR septic OR sepsis))))) OR ((TI (((Perinatal OR neonat^∗^ OR newborn OR new born) N2 (mortalit^∗^ OR death^∗^ OR infection^∗^ OR sepsis OR septic OR asphyxia OR jaundice OR fever^∗^ OR hypothermi^∗^ OR anaemi^∗^ OR anemi^∗^)).)) OR (AB (((Perinatal OR neonat^∗^ OR newborn OR new born) N2 (mortalit^∗^ OR death^∗^ OR infection^∗^ OR sepsis OR septic OR asphyxia OR jaundice OR fever^∗^ OR hypothermi^∗^ OR anaemi^∗^ OR anemi^∗^)).))) OR ((TI (((Maternal OR mother^∗^ OR pregnan^∗^) N2 (mortalit^∗^ OR death^∗^)))) OR (AB (((Maternal OR mother^∗^ OR pregnan^∗^) N2 (mortalit^∗^ OR death^∗^))))) OR ((TI (((emergency OR unplanned) N2 (caesarean OR cesarean OR c-section)))) OR (AB (((emergency OR unplanned) N2 (caesarean OR cesarean OR c-section))))) OR ((MH “Maternal Mortality”)) OR ((MH “Infant Mortality”)) OR ((MH “Infant Death”) OR (MH “Perinatal Death”)) OR ((MH “Puerperal Disorders”) OR (MH “Postpartum Hemorrhage”) OR (MH “Puerperal Infection”)) OR ((MH “Eclampsia”) OR (MH “Pre-Eclampsia”)) OR ((MH “Anemia, Neonatal”) OR (MH “Neonatal Sepsis”) OR (MH “Asphyxia Neonatorum”)) OR ((MH “Jaundice, Neonatal”))) AND (((MH “Time Factors”)) OR (TI (time OR timing)) OR (AB (time OR timing))))) AND (((TI (incidence OR prevalence OR epidemiolog^∗^ OR cohort^∗^ OR survey^∗^ OR cross-section^∗^ OR population OR observational OR quantitative)) OR (AB (incidence OR prevalence OR epidemiolog^∗^ OR cohort^∗^ OR survey^∗^ OR cross-section^∗^ OR population OR observational OR quantitative))) OR ((MH “Epidemiological Research”) OR (MH “Descriptive Research”) OR (MH “Health Services Research+”) OR (MH “Administrative Research”) OR (MH “Analytic Research”) OR (MH “Applied Research”) OR (MH “Clinical Research”) OR (MH “Survey Research”) OR (MH “Secondary Analysis”) OR (MH “Trend Studies”) OR (MH “Predictive Research”)) OR ((MH “Empirical Research”) OR (MH “Case Control Studies+”) OR (MH “Correlational Studies”) OR (MH “Cross Sectional Studies”) OR (MH “Prospective Studies+”) OR (MH “Retrospective Design”) OR (MH “Quasi-Experimental Studies+”) OR (MH “Repeated Measures”)))) “AND” “NOT” (((MH “Animals+”) OR (MH “Mammals+”)) “AND” “NOT” ((MH “Human”)))

## Appendix IV: Timing of overall postpartum maternal mortality and timing of cause-specific maternal mortality

Timing of overall maternal mortality with 95% confidence intervals (corresponds to Figure 2)

Timing of overall maternal mortality before 2010 and after 2011 with 95% confidence intervals (corresponds to Figure 3)

Timing of overall maternal mortality by country income classification with 95% confidence intervals (corresponds to Figure 4)

Timing of cause-specific maternal mortality with 95% confidence intervals (corresponds to Figure 5)
